# COVID-BEHAVE dataset: measuring human behaviour during the COVID-19 pandemic

**DOI:** 10.1038/s41597-022-01856-8

**Published:** 2022-12-06

**Authors:** Kostas Konsolakis, Oresti Banos, Miriam Cabrita, Hermie Hermens

**Affiliations:** 1 Biomedical Signals and Systems Research Group, Faculty of Electrical Engineering, Mathematics and Computer Science, University of Twente, Enschede, 7522NB The Netherlands; 2 Research Center for Information and Communication Technologies, University of Granada, Granada, E-18071 Spain; 3 Innovation Sprint, Drienerlolaan 5, Enschede, 7522NB The Netherlands

**Keywords:** Databases, Human behaviour

## Abstract

Aiming to illuminate the effects of enforced confinements on people’s lives, this paper presents a novel dataset that measures human behaviour holistically and longitudinally during the COVID-19 outbreak. In particular, we conducted a study during the first wave of the lockdown, where 21 healthy subjects from the Netherlands and Greece participated, collecting multimodal raw and processed data from smartphone sensors, activity trackers, and users’ responses to digital questionnaires. The study lasted more than two months, although the duration of the data collection varies per participant. The data are publicly available and can be used to model human behaviour in a broad sense as the dataset explores physical, social, emotional, and cognitive domains. The dataset offers an exemplary perspective on a given group of people that could be considered to build new models for investigating behaviour changes as a consequence of the lockdown. Importantly, to our knowledge, this is the first dataset combining passive sensing, experience sampling, and virtual assistants to study human behaviour dynamics in a prolonged lockdown situation.

## Abstract (summary)

**Table:** 

Measurement(s)	‘human behaviour during the COVID-19 outbreak’, ‘human behaviour’, ‘physical behaviour’, ‘social behaviour’, ‘emotional behaviour’, ‘cognitive behaviour’
Technology Type(s)	‘smartphone device’, ‘activity tracker’, ‘digital questionnaires’
Sample Characteristic - Organism	‘human beings’
Sample Characteristic - Location	‘The Netherlands’, ‘Greece’

## Background & Summary

In early 2020 the entire world witnessed an unforeseen transformation caused by the coronavirus disease 2019 (COVID-19) outbreak, forcing the World Health Organization (WHO) to declare it as a global health emergency^1^ and soon after as a pandemic^2^. To date, the COVID-19 pandemic has been responsible for over two hundred and fifty million reported cases worldwide, including more than five million deaths^3,4^ (as of 1 December 2021). Trying to restrict the spread of the COVID-19, citizens were asked to adhere to the lockdown policies and strict confinement measures followed by the governments around the world. The measures remained until early 2021, when the vaccines started becoming widely available and people got vaccinated progressively.

These unprecedented measures had an immediate impact on peoples’ lives, affecting dramatically their behaviour, their daily activities, and their lifestyle. During a pandemic, both social distancing and isolation might lead to high levels of perceived stress, resulting to poorer diet, physical inactivity, and reduced sleep duration and quality^5,6^. Furthermore, social isolation and loneliness can be harmful for mental health^7^. As a result, many researchers became aware of these consequences and started investigating the lockdown consequences on human health and mental well-being. Xiangyu Kong *et al*.^8^ reported the prevalence of depression and anxiety in hospitalised patients with COVID-19, the importance of social support, and the effect of psychological and behavioural interventions. Furthermore, many studies focused on the lockdown impact on people’s lifestyle and their potential behavioural changes. Many works conducted cross-sectional studies using online surveys and questionnaires to evaluate the changes on users’ daily lives (e.g., physical activity, diet, sleep, etc.)^9–12^. Additionally, researchers evaluated the effects of COVID-19 on human behaviour by using multimodal and unobtrusive sensing devices to collect real-world data, such as data from smartphones, wearable devices, and digital questionnaires^13–15^.

However, the effects of the restrictive measures caused by the COVID-19 outbreak on human behaviour have not been thoroughly investigated yet, and to the best of our knowledge, there is no study yet focusing on analysing the physical, social, emotional, and cognitive aspects of human behaviour at once and during the lockdown. Trying to shed some light on the COVID-19 pandemic outcomes and how an enforced lockdown affects people’s lives, we conducted a study for measuring human behaviour during the first wave of the COVID-19 lockdown (May-August 2020), when people still could not get vaccinated against the disease. The study focused on measuring both the physical, social, emotional, and cognitive dimensions of behaviour by collecting data from smartphones (such as the ambient noise, location, the number of incoming/outgoing phone calls, and the number of received/sent text messages), activity trackers, users’ input, and users’ demographics. In total, 21 healthy subjects managed to complete the data collection phase, allowing to acquire continuous records of data for more than two months. As such, the current study aims to provide longitudinal data covering human behaviour in a holistic way. The collected data can be used to investigate the possible effects of the lockdown on the study participants and how their behaviours change before, during, and after a lockdown that is imposed due to a pandemic.

Overall, the enforced lockdown due to the COVID-19 pandemic was a peculiar situation that may not take place anytime soon or ever again in order to further investigate human behaviours during a pandemic outbreak. Taking this into consideration, we decided to make the dataset available for open access, by providing longitudinal data related to measuring human behaviour during the COVID-19, focusing on physical, social, emotional, and cognitive behaviour aspects at the same time. Eventually, we believe that this study is of great importance to the scientific community since many researchers can consider using the dataset for further analysis, by digging into participants’ day-to-day lives during the lockdown. Thus, the current dataset could play a major role in understanding human behaviour, giving the opportunity to quantify variations of human behaviour, and especially, in response to specific measures and policies applied by the cause of an event, such as a pandemic outbreak.

## Methods

### Study design

The COVID-19 pandemic crisis had limited severely the options for recruiting participants since national regulations prohibited in general close contact (social distancing rules). This situation played a major role in setting up the experiment (such as distributing and configuring the sensing devices for the study participants). For this reason, and yet to ensure a reliable data collection, we decided to recruit healthy participants who already owned the necessary devices and could follow the instructions of the study remotely. Additionally, the study was conducted in the Netherlands and in Greece, providing a first insight into two European countries that followed different confinement measures to explore how these would affect the wellbeing of the residents. During the time the data collection took place, there was a hard lockdown in Greece followed by a strict curfew, while the lockdown rules imposed by the Dutch government were much softer. For instance, Dutch residents were allowed to go for a walk outdoors throughout the day, while Greek residents were restricted to stay at home and go out only for a valid reason (such as paying a visit to the doctor). Hence, the study was designed to take place during the COVID-19 lockdown, where we would have the opportunity to measure human behaviour during and after the lockdown, and presumably changes in between.

Participants were asked to use their own smartphone and activity tracker device (if any), as usual, and to answer a few online questions on a daily basis. An activity tracker is considered here any wrist-worn device (e.g., Fitbit, Garmin, Apple Watch) that tracks the performed activities or steps but also any pedometer mobile application that counts steps. The study was performed in uncontrolled settings, and thus, no explicit instructions were given to the participants on what tasks to perform or how often.

The study started after receiving the approval from the Ethics Committee EEMCS of the University of Twente (UT), with registration number ‘RP 2020–43’. The data collection namely started on Wednesday, May 20th, 2020 (the actual date varies per participant, since some subjects started a bit later depending on their availability) and it ended on Sunday, July 5th, 2020. However, there were some participants who decided, upon request, to continue with the data collection for a few more days. Some days later, a compensation (voucher for online shopping) was given to those participants who completed the study.

### Participants

The recruitment was performed based on convenience sampling, asking for participants who were owing the necessary devices and were willing to participate in the experiment during the COVID-19 outbreak. Therefore, we did not specify any recruitment criteria that could reflect specific societal aspects (such as socioeconomically, demographically, or through participant’s education/employment attainment). During the recruitment phase, an information brochure including a short description of the study was distributed online via the University of Twente’s social media (Facebook, Twitter, LinkedIn). Further explanations of the research study were given to the candidates, including participants’ tasks and data privacy considerations. The recruited participants had to confirm their participation by signing the informed consent form, while final instructions were given explaining how to set up the sensing devices in order to participate in the study.

Initially, more than 30 participants showed interest to participate in the study. However, only 23 confirmed to participate in the data collection phase. Two of them decided to withdraw in a later phase. Finally, 21 subjects aged from 25 to 58 years old (average age 32.30 ± 7.80 years) completed the data collection period. Among the participants, 15 were females (average age 31.70 ± 7.45 years) and 6 were men (average age 34.00 ± 9.00 years). Regarding the location and the users’ environment, 5 participants were located in Greece, while the rest were living in the Netherlands during the data collection phase. It is worth mentioning that among the participants there was one pensioner with low-back chronic pain, two healthy students, while the rest of the subjects were healthy adults (some of them were working remotely and some of them were visiting their office regularly). Eventually, the participants were divided into two groups (Group A and Group B) according to the devices they used for the data collection. An overview of the Group A and Group B participants can be seen on Table 1 and Table 2, respectively.

**Table 1:** Overview of the participants for Group A.

subject	sex	age	country	occupation	input	mobile	web	activity tracker	duration (days)
s01	female	58	Greece	pensioner	both	Android	yes	Fitbit	80
s04	male	29	Netherlands	employee	both	Android	yes	Fitbit	49
s09	female	30	Greece	employee	both	Android	yes	app	47
s12	male	29	Netherlands	employee	both	Android	yes	Fitbit	42
s13	female	30	Netherlands	employee	both	Android	yes	Fitbit	44
s14	female	37	Netherlands	employee	both	Android	yes	Fitbit	45
s15	female	30	Netherlands	employee	both	Android	yes	Fitbit	41
s17	female	33	Netherlands	employee	app	Android	no	other	34
s18	female	25	Netherlands	student	app	Android	no	Garmin	41
s19	female	30	Netherlands	employee	both	Android	yes	app	46
s20	male	54	Netherlands	employee	app	Android	yes	Fitbit	48
s21	male	30	Netherlands	employee	both	Android	yes	Fitbit	76

**Table 2:** Overview of the participants for Group B.

subject	sex	age	country	occupation	input	mobile	web	activity tracker	duration (days)
s02	female	30	Netherlands	employee	web	no	yes	Fitbit	48
s03	female	28	Greece	employee	web	iOS	yes	app	45
s05	female	29	Netherlands	employee	web	no	yes	app	46
s06	male	32	Netherlands	employee	web	iOS	yes	app	46
s07	female	29	Netherlands	employee	web	iOS	yes	app	50
s08	female	30	Greece	employee	web	no	yes	app	42
s10	female	28	Netherlands	employee	web	no	yes	app	41
s11	male	30	Greece	employee	web	no	yes	app	40
s16	female	29	Netherlands	student	web	iOS	yes	app	46

Group A consists of 12 participants (average age 34.58 ± 9.97 years). Ten of them were living in the Netherlands, while the rest were living in Greece. The collected data consist of samples acquired through the mobile app (running on Android OS devices), activity tracker (also including pedometer applications), mobile-based questions (via the mobile app), and web-based questions (via the website).

Group B consists of 9 participants (average age 29.44 ± 1.17 years), for whom the collected data include samples from the mobile app (running on iOS devices), activity tracker (also including pedometer applications), and web-based questions (via the website). Six of them were living in the Netherlands, while three were living in Greece. Furthermore, four participants from this group installed the mobile app on iOS devices, while the rest did not install the app on their smartphone device (either they did not manage to install the app by themselves, or they were sceptical due to data privacy concerns).

### Materials

The data collection, handling, and management were performed via the Behaviour Analysis Framework^16^ (a novel framework for sensing, identifying and quantifying different domains of human behaviour in a holistic way), applying the necessary technologies to collect, store, and maintain multimodal sensor data securely on the framework’s server. Respecting participants’ privacy, the collected data were anonymised and stored to a secure server at the University of Twente. All data were de-identified, and thus, neither real names nor private information was stored. Furthermore, data access was limited only to researchers with authorised credentials and passwords.

Before the data collection phase took place, the participants received two tutorials with detailed instructions on a) how to install the mobile app^17^ on their mobile Android or iOS devices and what smartphone sensors to enable/disable for the data acquisition with respect to the requirements of this study, and b) how to register and use the website^18^ in order to connect their activity tracker and answer the online questions.

The mobile app here refers to the AWARE app^17^ and it was used to collect data records from users’ smartphones and upload them on the UT server. Hence, sensor data related to accelerometer, GPS, ambient noise, phone calls, and text messages were collected and processed via this app. The AWARE app was also used to trigger the mobile-based questions, hereinafter referred to as the Experience Sampling Method (ESM) questions. The mobile app version used was the aware-phone-armeabi-release.apk published on 28 April 2020 for Android devices^19,20^ and the AWARE Client V2 1.10.9 published on 29 March 2020 for iOS devices^21,22^.

The website here refers to the Council of Coaches (COUCH) website^18^. This website was used by the participants to answer the online questions, which are presented to the users via a virtual coach instead of traditional prototypical questions to increase response adherence^23^. We refer to these as the Coach-as-a-Sensor (CaaS) questions. The CaaS represents any type of information that can be actively acquired during a conversation with a virtual character (e.g., emotional coach) and not passively through sensor data. During a virtual conversation, the user is asked some questions in order to monitor user’s behaviour. For example, a virtual coach expertise in emotional behaviour can ask CaaS questions about user’s mood (e.g., “*How are you feeling today? Could you describe your overall mood during the morning hours?*”) when the conversation allows it. Similarly, another coach expertise in cognitive behaviour can ask questions related to the engagement of the user with cognitive tasks (e.g., “*Have you read a book today?*”). Both of these questions aim to estimate user’s feelings, such as being sad (including answers for being very negative or negative), happy (including answers for being positive or very positive), or whether they were involved with cognitive tasks. Such questions are interspersed with the normal flow of the conversation, and in some cases, may be regarded as small talk or conversational connectors, thus reducing the perceived burden to the user compared to traditional ESM’s. However, our aim for this study was to use both ESM and CaaS questions as we believe they can complement each other rather than one being the replacement for the other.

In addition to the data collected via the Behaviour Analysis Framework, we also obtained data related to exogenous variables that could play a major role while analysing human behaviour. In particular, carrying out causality studies in human behaviour requires the consideration of confounding factors that could possibly force participants into certain choices. For instance, weather, news, social norms, and government measures could affect users’ behaviours. Depending on the granularity level of the exogenous variables, this can become a hardly approachable problem. In this work, we consider exogenous variables that refer to the weather conditions, but also to the outcomes of the pandemic outbreak in Greece and in the Netherlands. Meteorological data were obtained through the “*Meteostat*” repository^24^, referring to the weather conditions (e.g., temperature, precipitation, wind speed, and air pressure) in Athens and in Amsterdam for the period of the data collection. Moreover, a collection of data is provided via the “*Our World in Data*” database^4^, including observations related to the COVID-19 outbreak from January 2020 up to September 2020. These data refer to exogenous variables such as the number of confirmed cases and deaths, excess mortality, policy response, reproduction rate, tests and positivity, etc.

### Data collection

Initially, the AWARE app was installed on the user’s smartphone, which allowed the smartphone data acquisition and the triggering of the mobile-based questions. The collected smartphone data consist of accelerometer and GPS signals for tracking physical activity, while ambient noise, location, the number of incoming/outgoing phone calls, and the number of received/sent text messages can be used to detect the user’s level of being socially active.

GPS coordinates are used to cluster the locations that a person visited throughout the day (e.g., home, work office, supermarket, etc.), estimating how much time the subjects spent at these locations (e.g., 20 hours at home). Clusters are calculated based on the DBSCAN (Density-Based Spatial Clustering of Applications with Noise) implementation, where 0.5 km is the maximum distance that points can be from each other to be considered in a cluster and 2 is the minimum cluster size (everything else is classified as noise with the value −1). Furthermore, the distance and travel speed are calculated for each detected location according to the Haversine distance between two arrays of points on the earth (specified in decimal degrees). The sampling frequency is every 180 seconds.

Apart from gathering raw data (such as GPS), some higher-level features are also collected. These data are acquired via AWARE plugin tools or are retrieved directly from the vendor’s server (e.g., Fitbit Inc.). Users’ physical activities have been detected and retrieved via the AWARE Plugin: Google Activity Recognition^25^, which is used to estimate a new activity inference among the following labels: walking, running, biking, being in a vehicle (car, bus), tilting, or unknown. The frequency range to detect a new activity varies in seconds, depending on the duration of the ongoing activity. This information can be used to capture users’ mode of transportation or estimate the intensity level of the performed activities (e.g., the activities running and biking can be clustered as vigorous activities, while being in a vehicle can be clustered as sedentary activities).

Additionally, activity trackers data are collected and include the number of steps and the intensity of the performed activities per day (i.e., sedentary, lightly active, fairly active, and very active), which can be used to enhance the understanding of users’ physical behaviour. This information is retrieved directly from the vendor’s server (e.g., Fitbit Inc.) or is manually annotated by the user and based on the user’s pedometer mobile application. The frequency for counting steps ranges from hours to days, depending on the source. For instance, the Fitbit data count steps every minute, while the pedometer apps data refer to steps per day.

Conversation data are collected through the microphone sensor and are used to analyse the ambient noise and estimate if there is an ongoing conversation. This information is retrieved via the AWARE Plugin: Audio Conversations^26^ to detect if the user is engaged or not in a conversation with one or more people, without storing raw audio. More specifically, the plugin follows a duty cycle of collecting audio for 1 minute, followed by 3 minutes pause, assuming that a real conversation can be detected in a time frame of 4 minutes (since a continuous audio recording would have a huge impact on the device battery drain).

Phone calls data refer to the time that an incoming or outgoing call is initiated, including its duration. Furthermore, each contact is characterised by a unique device id number, and thus, more features can be extracted. For instance, if the calls were performed between familiar persons (such as family members and friends) or between new unknown contacts.

Text messages data refer to the time that an SMS was sent or received. Similar to the phone calls feature, each contact is characterised by a unique device id number, so it can be investigated if the SMS were exchanged between familiar persons (such as family members and friends) or between new unknown contacts. For instance, a text message that is occasionally received by a sender, to whom the user does not answer, could indicate that these types of messages are random and do not actually reveal reliable knowledge about the user’s social behaviour (e.g., messages related to advertisement purposes).

Concerning the smartphone audio (e.g., ambient noise and phone calls) and text messages (SMS), these were only used for annotating if a user was socially active, without examining the audio or text content (no raw audio or text content were stored). For instance, the audio decibels were used for modelling user’s social behaviour (instead of the audio content) and detecting if a conversation was taking place. In addition to protecting users’ privacy, GPS data are processed accordingly so that the actual users’ GPS trajectories cannot be revealed (such as the coordinates of users’ home).

Furthermore, ESM and CaaS data have been collected, by asking the related questions on a daily basis through the mobile app and through the website, respectively. These questions can be used to monitor user’s social, emotional, and cognitive behaviour changes during and after the lockdown. The related answers consist of free text, numeric values, Likert scale, yes/no queries, and multiple choices and refer to user’s physical activities, social interactions, feelings, and cognitive tasks. The sampling frequency varies per question and per participant.

ESM data consist of users’ input on the questions triggered via the mobile app, related to the social, emotional, and cognitive behaviours. The questions (Q1-Q3) were triggered via a pop-up notification three times per day, at 12 pm, 5 pm, and 9 pm, referring to users’ behaviour during the morning, afternoon, and evening hours respectively. However, these questions were only answered by the participants from Group A, by using the mobile app on their Android device. Group B participants who installed the AWARE mobile app on iOS devices could not answer the ESM mobile-based questions since these were not supported at the data-collection time. An overview of the ESM questions can be seen on Table 3.

**Table 3:** An overview of the ESM mobile-based questions asked through the mobile app.

Measured Behaviour	Frequency	Question	Answer
Emotional	Three times per day: at 12 pm, 5 pm and 9 pm	**Q1**: How are you feeling right now?	5-Scale ranging from very sad to very happy
Social	Three times per day: at 12 pm, 5 pm, and 9 pm	**Q2**: Have you interacted with other people (physically or digitally), during the previous hours^1^? If so, please indicate the total duration in hours (rounding up).	5-Scale ranging from zero hours to five hours
Cognitive	Three times per day: at 12 pm, 5 pm, and 9 pm	**Q3**: Have you participated in any cognitive activities during the previous hours^1^? If so, please indicate the total duration in hours (rounding up). Cognitive activities are considered tasks, such as reading a book or newspaper, learning a new skill, watching an educational TV show or playing a board game (e.g., chess, Sudoku, etc.).	5-Scale ranging from zero hours to five hours

^1^Depending on the time frequency that the question was triggered, previous hours refer to either the morning hours (between 7am and 12 pm), or the afternoon hours (between 12 pm and 5 pm), or the evening hours (between 5 pm and 12am).

CaaS data consist of users’ input on the online questions triggered via the website, related to the social (Q4-Q8), emotional (Q9-Q11), and cognitive (Q12-Q16) behaviours. These questions were answered once per day based on the user’s availability and time preference, just by logging on the COUCH website^18^ and following the given instructions. An overview of these questions can be seen on Table 4.

**Table 4:** An overview of the CaaS web-based questions asked through the website.

Measured Behaviour	Frequency	Question	Answer
Social	Once per day	**Q4**: Are you satisfied with your social interactions today?	Multiple choice including very dissatisfied, dissatisfied, satisfied, very satisfied
	Once per day	**Q5**: Have you interacted physically or digitally with other people today?	Yes^2^ or No
	Once per day	**Q6**: Could you tell me the total duration (in minutes) of interacting with family members? So, for example, write “30” if you spent half an hour with family members, or “0” if you haven’t seen them.	Numeric input
	Once per day	**Q7**: Could you tell me the total duration (in minutes) of interacting with friends? Again, write “0” if you haven’t seen any.	Numeric input
	Once per day	**Q8**: Finally, could you tell me the total duration (in minutes) of interacting with other acquaintances (like colleagues)? Again, write “0” if you haven’t had any interaction.	Numeric input
Emotional	Once per day	**Q9**: Please describe your overall mood, from negative to positive, during the morning hours, let’s say between 8am and 12 pm.	Multiple choice including very negative, negative, neutral, positive, very positive
	Once per day	**Q10**: Now, please describe your overall mood, from negative to positive, during the afternoon hours, so between 12 pm and 5 pm.	Multiple choice including very negative, negative, neutral, positive, very positive
	Once per day	**Q11**: And finally, please describe your overall mood, from negative to positive, during the evening hours, so between 5 pm and 12am.	Multiple choice including very negative, negative, neutral, positive, very positive
Cognitive	Once per day	**Q12**: Have you participated in any of the following tasks today, such as reading a book or newspaper, learning a new skill, watching an educational TV show or playing a board game?	Yes^3^ or No
	Once per day	**Q13**: That’s very good. Now, could you tell me for how long did you spend reading today in minutes? Just type “0” if you didn’t read at all.	Numeric input
	Once per day	**Q14**: Okay, and how much time did you spend on learning a new skill, such as studying a foreign language, in minutes? Again, just type “0” if you did not do this today.	Numeric input
	Once per day	**Q15**: Very good! Now, how much time did you spend watching an educational TV-show? Again, just type “0” if you did not do this.	Numeric input
	Once per day	**Q16**: And finally, how much time did you spend either playing Sudoku, crossword games, puzzles, or other similar games in minutes?	Numeric input

^2^If the answer is Yes, then the following questions Q6-Q8 will be asked. Otherwise, they will be skipped.

^3^If the answer is Yes, then the following questions Q13-Q16 will be asked. Otherwise, they will be skipped.

A few additional questions (Q17-Q23) were asked on a weekly basis and at the end of the experiment. These questions were asked for special events or situations that occurred to the participants during the data collection, aiming to reflect significant changes in their behaviour. This users’ input contains valuable information since it can be used to evaluate the models for detecting behaviour changes that occur during the lockdown and how these changes differentiate when the users progressively return back to their normal lifestyle. An overview of these questions can be seen on Table 5. The questions Q17 and Q18 were asked at the end of each week to track any possible behaviour changes that followed on a weekly basis. Furthermore, the subjects were asked to answer the questions Q19-Q23 at the end of the data collection phase to identify possible behaviour changes during the lockdown period, and answer retrospectively if there were any significant changes in user’s behaviour before and after the COVID-19 lockdown. These questions were asked based on users’ preferences, either via the AWARE app or through online Google forms (for those who did not install the AWARE app).

**Table 5:** An overview of the questions related to the self-reported weekly events.

Measured Behaviour	Frequency	Question	Answer
User’s feedback for the last 7days	Every Sunday at 4 pm	**Q17**: Overall, how do you feel about the last 7 days?	5-Scale ranging from very dissatisfied to very satisfied
User’s feedback for the last 7days	Every Sunday at 4 pm	**Q18**: Try to compare this week (last 7 days) with the previous week. Did you experience any significant difference? If so, please specify. For example, you can type “more/less physically active”, “more/less socially active”. Furthermore, you can type anything that you think is relevant for this week; such as “no difference”, “this week I was tired/sick”, “this week I met more/fewer people”, etc.	Free text
General information related to the lockdown	Once, after the data collection phase	**Q19**: Depending on each country, the government announced some rules/suggestions for the end of the COVID-19 lockdown. You can consider that the lockdown was over for you when you started going out more frequently (going shopping, going to work and not working from home, going out with friends, etc.). Please estimate the date that the lockdown was over for you.	Free text
User’s feedback for estimating physical behaviour changes during the lockdown	Once, after the data collection phase	**Q20**: Try to compare the period during and after the lockdown. Did you experience any significant changes in your physical behaviour? If so, please specify. You can type anything that you think is relevant. For example, you can type “During the lockdown, I was walking every day because I had more free time”, “After the lockdown, I started going to the gym more frequently”, etc.	Free text
User’s feedback for estimating social behaviour changes during the lockdown	Once, after the data collection phase	**Q21**: Try to compare the period during and after the lockdown. Did you experience any significant changes in your social behaviour? If so, please specify. You can type anything that you think is relevant. For example, you can type “During the lockdown, I wasn’t satisfied with my social life and I felt lonely”, “After the lockdown, I try to go out more often and meet more people”, etc.	Free text
User’s feedback for estimating emotional behaviour changes during the lockdown	Once, after the data collection phase	**Q22**: Try to compare the period during and after the lockdown. Did you experience any significant changes in your emotional behaviour? If so, please specify. You can type anything that you think is relevant. For example, you can type “During the lockdown, I was sad and stressed due to the virus pandemic”, “After the lockdown, I feel happier because I can travel again”, etc.	Free text
User’s feedback for estimating cognitive behaviour changes during the lockdown	Once, after the data collection phase	**Q23**: Try to compare the period during and after the lockdown. Did you experience any significant changes in your cognitive behaviour? If so, please specify. You can type anything that you think is relevant. For example, you can type “During the lockdown, I was reading more books”, “After the lockdown, I play board games with friends more often”, etc.	Free text

### Limitations

The current dataset fairly represents some of the behaviours observed during and after the first wave of the COVID-19 lockdown, and their presumably changes in between. Even though we firmly believe that this study serves fully to the task of helping researchers to develop and test novel methodologies to study human behaviour individually and collectively, there is a number of limitations that need to be considered. Apart from typical cultural and ambient differences, the restrictions posed by governments during the lockdown differed among countries and even in between regions. Furthermore, the sample size is rather limited to generalise conclusions, and thus, the dataset should not be used to extrapolate further learning at societal level.

The strict confinement measures due to the COVID-19 outbreak hampered the recruitment phase. Therefore, the recruitment and the set-up of the experiment took place online based on convenience sampling, asking for participants who owned the necessary devices and were willing to participate in the experiment during the COVID-19 outbreak. This was a real barrier especially for senior participants, since we could not meet them in person and help them, for example, install the data collection app. It should be also noted that some people raised understandable concerns given the nature of the collected data, despite the fact that all data are collected and treated anonymously. Some individuals eventually accepted to participate after clarification, yet others declined the invitation for not being able to talk this through in person. Consequently, the strict restrictions posed by the COVID-19 lockdown had an inevitable impact on the recruited participants and their sample distribution in terms of regular factors (e.g., participants’ age, gender, income, etc.). Therefore, the collected data should not be used to reflect communities based on certain criteria, such as socioeconomically or demographically.

The data collection was conducted based on users’ availability and preferences, but also based on the available sensing devices that they owned. That means that different types of data were collected among participants. Trying to ensure data quality and integrity, we divided the participants into two groups: Group A and Group B. For Group A the data collection was conducted without any restrictions. However, the data related to Group B were not always consistent, since different types of data were collected among participants. For instance, some participants were not able to install the mobile app on their smartphones, while others did not give consent to allow access on retrieving their own smartphone data. Specifically, 5 participants (i.e., s02, s05, s08, s10, and s11) used only the activity tracker and answered the web-based questions, while only 4 participants from Group B gave consent to collect mobile data.

Another important remark is that the participants who installed the mobile app on iOS devices could not answer the ESM mobile-based questions due to the limitations on the iOS app (this option was not supported at the time of the data collection). Furthermore, we noticed that there is a significant amount of missing data related to the CaaS and/or the mobile sensor recordings for some subjects (e.g., s05, s13 and s15). The former could be due to the fact that subjects might be forgetting to log-in on the web-browser and answer the CaaS questions on a daily basis. The latter could be because either the subjects were accidentally disabling the mobile app to run in the background and operate smoothly (i.e., record sensor data and trigger ESM questions continuously), or the device settings for the battery optimisation were not fully adjusted to permanently allow the app to run in the background, resulting to discontinuous sensor recordings.

Depending on the smartphone manufacturers, some Android devices have a strict policy for stopping apps that run in the background after a certain time due to the battery optimisation. This ‘sleeping mode’ function ensures that unnecessary apps that run in silent mode will not affect the battery life of the smartphone device. However, this function could disable the mobile app from registering sensor data, continuously. Before the data collection started, we informed the participants on how to disable this ‘sleeping mode’ function, by providing a thorough set of instructions according to their device model. Additionally, the study investigator had a private video call with some participants for further assistance. However, a few participants found these instructions still hard to follow, since they were not familiar with adjusting the settings on their device, and eventually did not manage to successfully disable the battery optimisation for the AWARE app. Consequently, the mobile app was occasionally entering the ‘sleeping mode’ and the subjects had to open again the AWARE app for enabling it to run in the background, collect and upload data on the server.

While most of the collected data required a preprocessing phase to get them cleaned and ready for dissemination, the mobile data (apart from the GPS coordinates that required further processing to ensure users’ anonymity) were automatically processed and cleaned through the AWARE mobile app. For instance, physical activities and conversation data were automatically processed via the AWARE integrated plugins to provide the necessary information (e.g., Google Activity Recognition and Audio Conversations). However, some of the automatically processed recordings might contain noise due to the nature of the technology (e.g., the transition from Wi-Fi to cellular network could cause some inconsistencies), depending also on the smartphone manufacturer and the device permissions to record these data. For instance, GPS data (such as travel speed and distance features) for s01 consist of some nearly impossible values that do not always represent the actual movement. Another limitation of the GPS data is that real coordinates could not be made publicly available, and therefore, we calculated the difference of longitude and latitude, row by row, from their previous recordings. Consequently, we recommend further cleaning for the acquired data related to GPS, physical activities, and conversation.

## Data Records

The collected data are made available for public access via the *Open Science Framework (OSF)* repository Konsolakis:COVID-BEHAVE:2021^27^. The dataset includes a questionnaires description, associated metadata, and documentation, and follows the FAIR principles and the recent RDA guidelines for COVID-19-related datasets^28^. The data have been stored in three comma-separated values (CSV) files that are described in detail below.

### Participants’ characteristics

The provided information related to the participants demographics, and the types of sensing devices that they used, is stored in the file participants.csv. It is worth mentioning that s15 bought a new Android device phone during the data collection, and thus, two device ids are registered for this user, subsequently. An overview of the participants’ characteristics can be seen on Table 1 (Group A) and Table 2 (Group B).

**Fig. 1:**
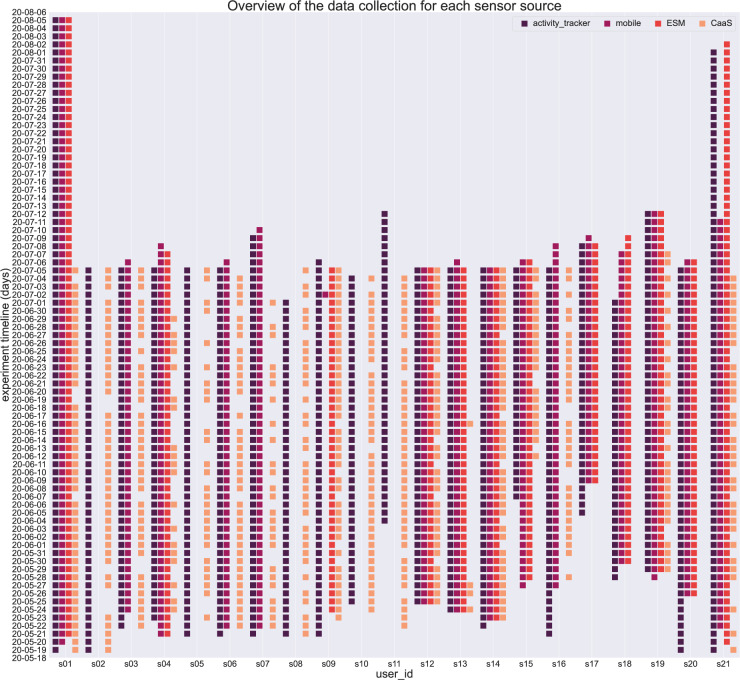
Timeline of the data collection for each sensor source and subject.

An overview of the overall data collection for each subject is shown in Fig. 1. This figure shows a timeline that represents what types of data have been collected per day and per subject. In particular, an overview of the data collection for each source is depicted, referring to the data acquired from activity trackers, mobile devices, ESM, and CaaS questions. It is clear that the sampling frequency varies per sensor source and per participant. However, we can see that most of the participants provided data for approximately 6 weeks, while s01 and s21 provided data for more than 2 months.

### Steps

**Fig. 2:**
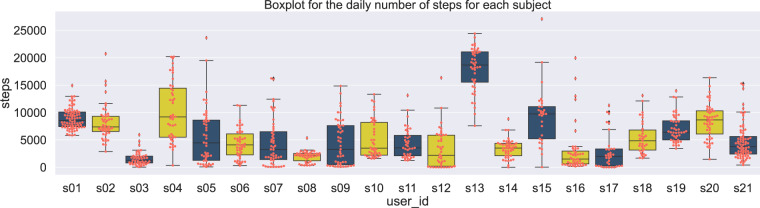
Data distribution of the daily number of steps across subjects.

The total number of steps is provided for each user and is stored in the file steps All Subjects.csv. The file is located in the folder activity tracker/steps. The data distribution for the steps is depicted in Fig. 2, where the boxplots represent the variation of the total number of steps per day and per subject.

### Physical activities & intensity

**Fig. 3:**
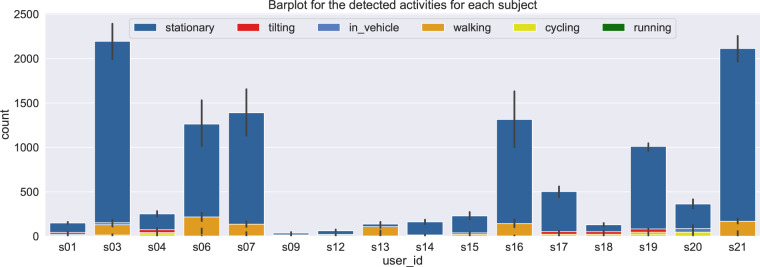
Data distribution of all the physical activities across subjects, including the related error bars (with 95% confidence interval).

**Fig. 4:**
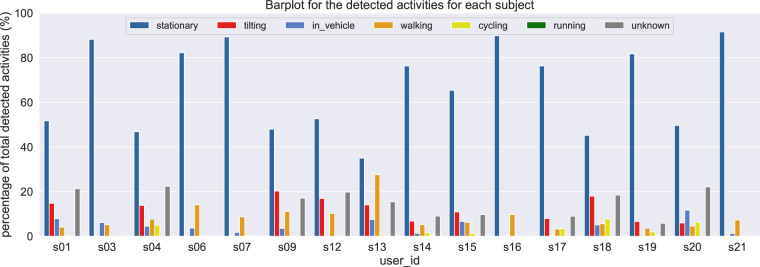
Data distribution of all the physical activities across subjects, based on the percentage values of the total number of detected activities.

The physical activities data are stored in the file AR All Subjects.csv, located in the folder mobile data/activity. Physical activities data refer to the performed activities (i.e., walking, running, biking, being in a vehicle, tilting or unknown) that were acquired for certain subjects and based on their smartphone devices. The distribution for all the detected activities can be seen in Fig. 3 and Fig. 4, where the bar plots compare the total number of the detected activities per subject. In particular, Fig. 3 aims to highlight the unbalanced data points among subjects for the different performed activities, while Fig. 4 aims to depict the total percentage of the detected activities for each subject.

**Fig. 5:**
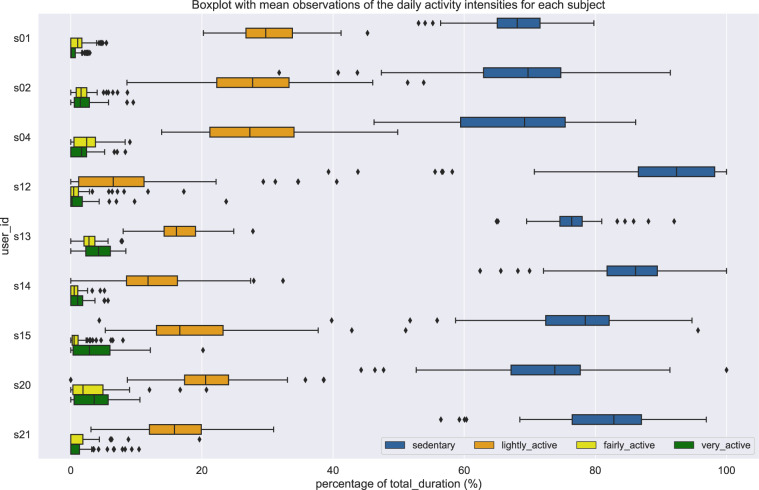
Data distribution for the intensity of the daily activities across subjects.

The intensity data are stored in the file intensity All Subjects.csv. The file is located in the folder activity tracker/activity intensity. The intensity data are acquired from Fitbit activity trackers and contain information regarding the intensity of the performed activities per day, and especially the total duration for being sedentary, lightly active, fairly active, and very active. The distribution for the activity intensities is depicted in Fig. 5, where the percentage of the intensity duration is plotted per day and per subject.

### Location

**Fig. 6:**
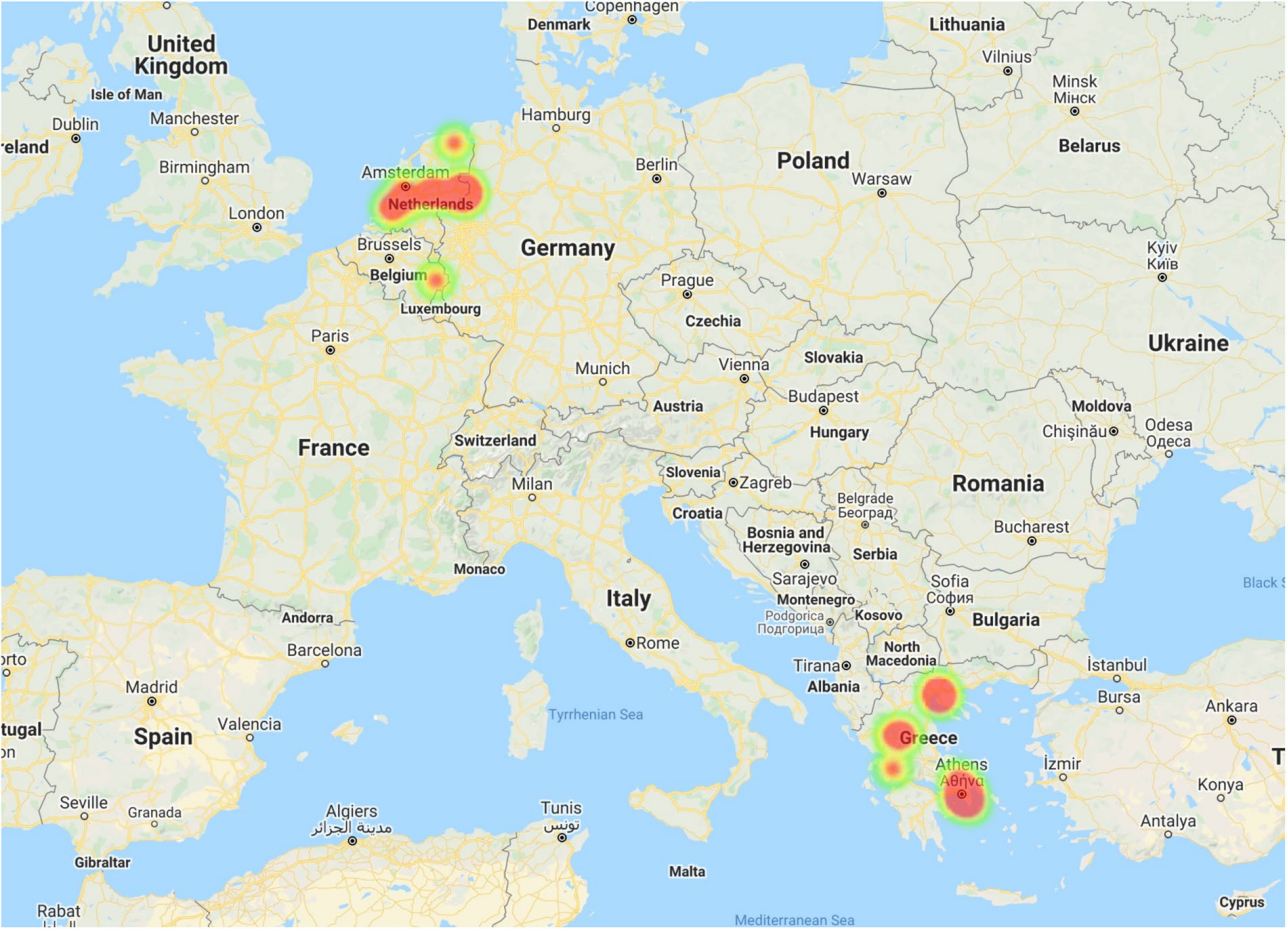
Geographical distribution across participants.

The GPS data are stored in the file GPS All Subjects.csv. The file is in the folder mobile data/GPS and refers to the difference of the GPS coordinates (the difference of latitude and longitude compared to the previous registered values), the number of clustered locations, the distance and the travel speed per user. The spatial representative points of the most visited locations are depicted in Fig. 6. For instance, we can see the regions that the subjects were located in the Netherlands and in Greece. Additionally, we can see that a subject travelled to Belgium during the data collection.

### Conversation

**Fig. 7:**
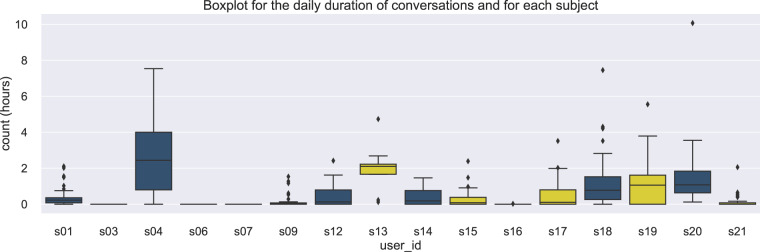
Data distribution of an ongoing conversation across subjects.

The conversation data are stored in the file audio All Subjects.csv, located in the folder mobile data/conversations. This file refers to the ambient audio of an ongoing conversation and were acquired based on the microphone sensor from smartphone devices. In Fig. 7, the boxplot distribution is depicted for every participant in the range of hours. For the iOS app users (s03, s06, s07, and s16) no actual conversation were detected. For these participants, the double energy feature could be a better estimator for estimating if there is an ongoing conversation.

### Phone calls

**Fig. 8:**
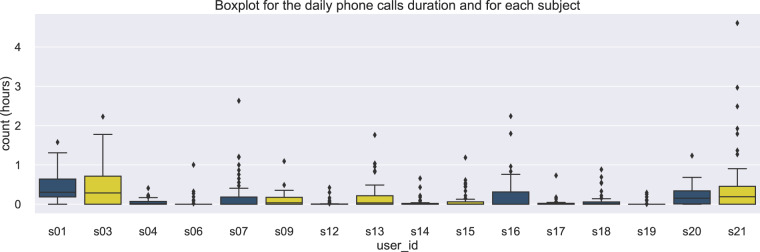
Data distribution for the duration of incoming/outgoing phone calls across subjects.

The phone calls data are stored in the file calls All Subjects.csv, located in the folder mobile data/calls. These data refer to the time that an incoming or outgoing call is initiated, including its duration. The time distribution (duration in hours) of an incoming /outgoing phone call can be seen for all the related participants in Fig. 8.

### SMS

**Fig. 9:**
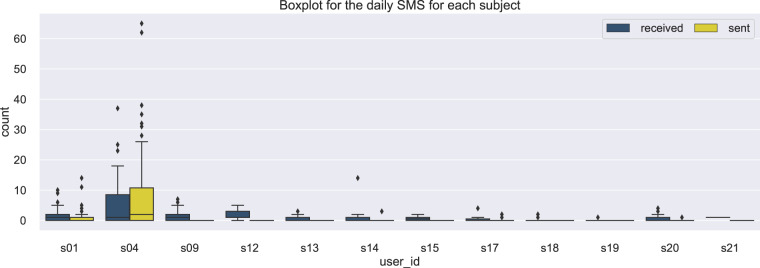
Data distribution of receiving or sending an SMS across subjects.

The text messages data are stored in the file SMS All Subjects.csv, located in the folder mobile data/SMS. They refer to the time that an SMS was sent or received. In Fig. 9 the boxplot distribution of receiving or sending an SMS is depicted for all subjects.

### Users’ input

**Fig. 10:**
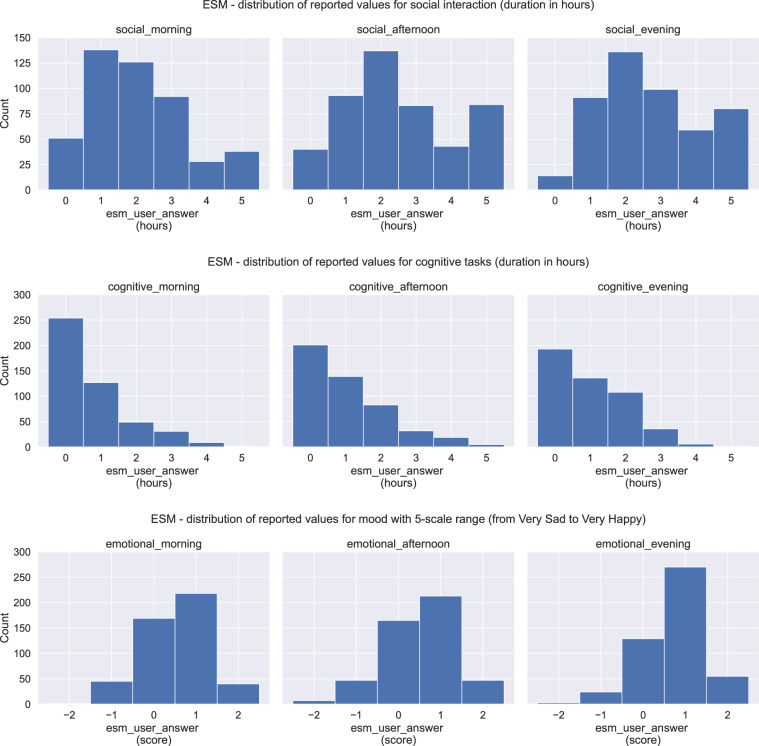
Data distribution of ESM user’s input related to social, cognitive and emotional answers.

**Fig. 11:**
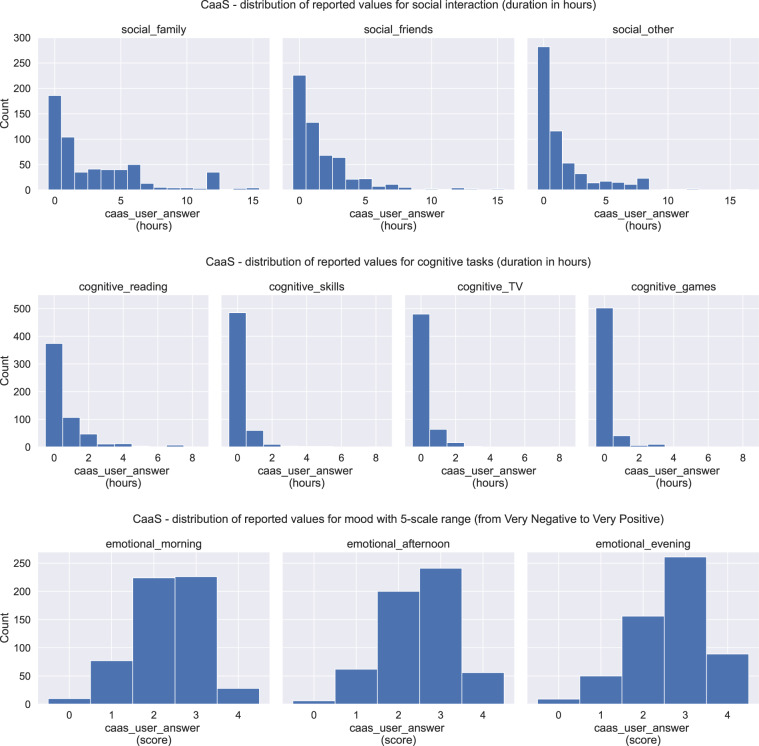
Distribution for CaaS user’s input related to social, cognitive and emotional answers.

**Fig. 12:**
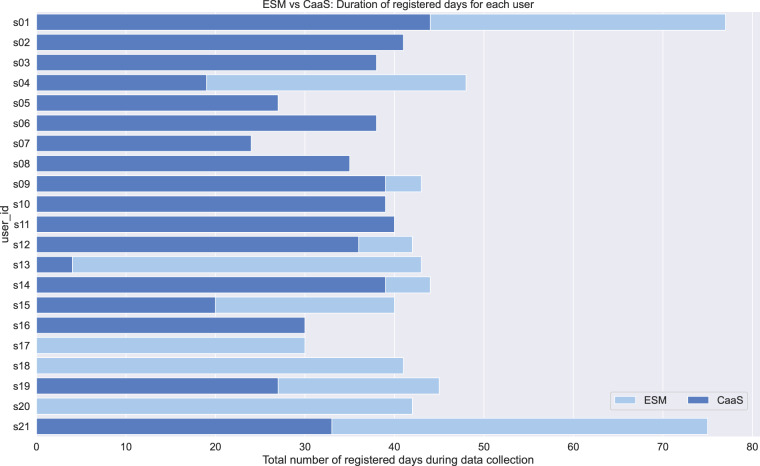
Registered days of collecting data related to ESM and CaaS users’ input.

Users’ input data refer to users’ answers on the ESM and CaaS questions. The ESM data are stored in the file ESM All Subjects.csv, located in the folder ESM. In Fig. 10, the ESM distribution is depicted for all the related ESM questions, including the histograms for the social, cognitive, and emotional values for the morning, afternoon, and evening hours. The CaaS data are stored in the file CaaS All Subjects.csv, located in the folder CaaS. In Fig. 11 we can see the CaaS data distribution for all the related questions, including the social, cognitive, and emotional user’s input. Compared to the ESM data distribution, we can see that the two figures show more differences than similarities as someone would expect. This is caused by the fact that only Group A participants (9 in total) answered the ESM questions, while the CaaS questions were answered by almost all the participants from both Group A and Group B, excluding the participants s17, s18 and s20 (that is 18 participants in total). For further explanation see also Fig. 12, which illustrates the total registered days of answering the ESM compared to the CaaS questions.

### Self-reported weekly events

**Fig. 13:**
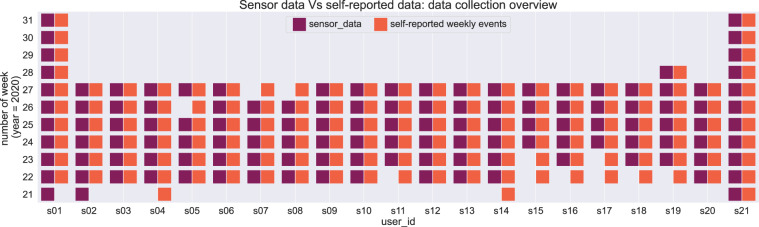
Data collection overview for self-reported weekly events.

The data related to the self-reported weekly events are stored in the file input All Subjects.csv, located in the folder weekly input. An overview of the collected sensor data compared to the self-reported weekly events data is depicted in Fig. 13. Here, the timeline is plotted in the range of weeks. A week with valid sensor data is considered a week where at least five days are registered with data from activity trackers and mobile devices, combined with data from the ESM questions and/or the CaaS questions. Similarly, we can see that most of the participants provided sensor and self-reported data for 6 weeks, while s01 and s21 provided data for more than 10 weeks.

### Exogenous variables

The exogenous variables here refer to the data obtained via the “*Meteostat*” repository^24^ and the “*Our World in Data*” database^4^. These are the weather conditions and the policy measures adopted in Greece and in the Netherlands during the pandemic outbreak. In particular, the meteorological data provide information related to the temperature, precipitation, wind speed, and air pressure, based on their capital cities (i.e., Athens and Amsterdam). These are stored in the folder exogenous variables/weather. Furthermore, the observations related to the COVID-19 outbreak and the governments response are stored in the folder exogenous variables/government response tracker. These data cover countries’ profile during the pandemic, presenting information about the number of confirmed cases and deaths, the number of conducted tests and positivity, excess mortality, government measures and response, and reproduction rate.

## Technical Validation

To ensure data robustness and obtrusiveness, we conducted a pilot study before the actual data collection took place. We confirmed that all data recordings could be successfully stored and retrieved via the UT server, and that the mobile app could be successfully installed on various mobile Android and iOS devices.

Furthermore, subjects were linked to a unique device id, ensuring that each user could only upload data from one device. In addition to this, subjects had to login with their credentials to the website and answer the CaaS questions. Since these questions represent users’ input retrospectively, we asked them to answer the CaaS questions daily and after 6 pm. This happened to prevent inserting incorrect information (e.g., answering at 11am how much time they spent with family for this day). In particular, the user had the option to login and answer the questions at multiple times per day (after 6 pm). For instance, the user could answer the social related questions at 9 pm, then the emotional related questions at 10 pm, and then the cognitive related questions at 11 pm. However, we disabled the option of answering more than once the same question and for the same day. For instance, a user who answered the social related questions at 10 pm was not allowed to answer again the same questions later this day. This happened to prevent double registrations. There was an option though to edit the answers before clicking on the final submission button for each question, in case the user accidentally inserted a wrong input.

Additionally, the study investigator was checking the database every day to ensure data reliability and to notify subjects in case there was an issue with the data recordings from the mobile app. This could happen because either the subjects were accidentally disabling the mobile app to run in the background, or the device settings for the battery optimisation were not successfully adjusted to allow the app to run in the background. In that case, we were contacting the participants to re-enable the mobile app to run in the background and operate smoothly by recording sensor data and triggering ESM questions continuously. Moreover, the study investigator was checking the database to identify any possible duplicate records. In the rare case that this might have happened, we considered data from the same subject that were registered with the same timestamp as duplicates and kept only the first registrations.

## Usage Notes

The dataset, metadata and documentation are publicly available for research purposes in the *OSF* repository Konsolakis:COVID-BEHAVE:2021^27^. No request is required for data download. Columns in each CSV file are delimited by semicolons.

## Data Availability

No custom code was used to generate or process the data described in the manuscript.
